# Bowels

**Published:** 1869-08-15

**Authors:** 


### *Bowels.*

Some of our correspondents wish to know whether, as the newspapers report, a certain irregular practitioner of this city, did, in point of fact, recently, in operating for strangulated hernia in a woman, remove four feet and some odd inches of intestine, with recovery of the patient without any unpleasant symptoms.

The thing is possible. Over thirty years ago the writer was present at an operation of the kind, when some eighteen inches of sphacelated intestine were removed, an artificial anus first established, and the continuity of the intestine subsequently established by a secondary procedure. Many similar cases, and some of a still more extraordinary character, are of record.

In the newspaper case in question there are several elements of improbability. So large a hernial protrusion, in a *woman*, unless of the umbilical variety, is exceedingly unlikely, and "the identical gut" being in a bottle at the operator's office, does not materially enhance the value of his assertion of where it came from. While belief is in suspense, we have arising in memory an alleged fracture of a patella into six pieces, which was cured in a brief period under treatment, one principal feature of which was a double inclined plane.

